# Simultaneous Supplementation of *Bacillus subtilis* and Antibiotic Growth Promoters by Stages Improved Intestinal Function of Pullets by Altering Gut Microbiota

**DOI:** 10.3389/fmicb.2018.02328

**Published:** 2018-10-12

**Authors:** Xueyuan Li, Shengru Wu, Xinyi Li, Tao Yan, Yongle Duan, Xin Yang, Yulan Duan, Qingzhu Sun, Xiaojun Yang

**Affiliations:** ^1^College of Animal Science and Technology, Northwest A&F University, Yangling, China; ^2^College of Animal Science and Technology, Hebei Agricultural University, Baoding, China

**Keywords:** early nutrition, pullets, intestinal morphology, 16S rRNA, gut microbiota

## Abstract

Early nutrition of pullets could determine the overall development and the performance of laying hens. With the aim to reduce the use of antibiotic growth promoters (AGPs) and to maintain the growth and development of pullets, the effect of simultaneous short-termed supplementation of AGPs (bacitracin zinc 20 mg/kg and colistin sulfate 4 mg/kg) and *Bacillus subtilis* (*B. subtilis*) DSM17299 probiotic, as well as the effect of supplementation of AGPs (bacitracin zinc 20 mg/kg and colistin sulfate 4 mg/kg) during the whole period (0~16 weeks) on the overall growth and development, intestinal health, and caecal microbiota of pullets were evaluated. In the present study, a total of 630 one-day-old Hy-Line Brown layers were randomly distributed into five equal groups: including the AGPs group (supplemented with AGPs based on basal diets for 16 weeks), the BA3 group (supplemented with AGPs and *B*. *subtilis* based on basal diets for 3 weeks), the BA6 group (for 6 weeks), the BA12 group (for 12 weeks), and the BA16 group (for 16 weeks). When compared with the AGPs group, the supplementation of AGPs + *B. subtilis* for the first 3 weeks could maintain overall growth performance, including the average body weight, average feed intake, average daily weight gain, and feed conversion ratio of pullets at 3, 6, 12, and 16 weeks of age (*P* > 0.05). Meanwhile, the characteristic growth indexes in different periods were separately measured. At 3 weeks of age, the amylase activity in ileum was elevated (*P* = 0.028), and the length of tibia was up to the standard in the BA3 group. At 12 weeks of age, the increased villus height (*P* = 0.046) of jejunum, increased villus height (*P* = 0.023) and ratio of villus height to crypt depth (*P* = 0.012) of ileum, decreased crypt depth (*P* = 0.002) of ileum, and elevated mRNA levels of sucrase in jejunum (*P* < 0.05) were all identified in the BA3 group. At 16 weeks of age, the secreted immunoglobulin A (sIgA) content in the jejunum mucosa of the BA3 group was greater than the other groups (*P* < 0.001). Furthermore, altered intestinal microbiota was found in the BA3 group. Specifically, decreased amounts of *Alistipes, Bacteroides, Odoribacter, Dehalobacterium*, and *Sutterella* and increased amounts of *Lactobacillus, Dorea, Ruminococcus*, and *Oscillospira* were determined (*P* < 0.05) in the BA3 group at week 6. Meanwhile, decreased amounts of *B. fragilis* and *C. leptum* (*P* < 0.05) were identified in the BA3 group at week 12, which were found to be relevant for the improvement of intestinal morphology (*P* < 0.05) by Pearson analysis. In conclusion, simultaneous supplementation of AGP and *B. subtilis* for 0~3 weeks increased the relative abundance of beneficial microbiota in caecum in 0~6 weeks, then improved the intestinal morphology by elevating populations of *B. fragilis* and *C. leptum* in 7~16 weeks, and further upregulated sucrase expression and increased sIgA content in the intestinal mucosa in 13~16 weeks.

## Introduction

Antibiotic growth promoters (AGPs) are widely used in livestock production to provide protection against pathogens and to maintain health and productivity (Brown et al., 2017). However, the use of AGPs in animal feeding is often argued upon because of the issues of antimicrobial resistance and drug residue, as well as its potential harmful effect on intestinal microbial homeostasis (Van Boeckel et al., 2015). With the increase in demand for poultry products with high quality, it is imperative to exploit effective special additives and related additive approach that can both stimulate the productive potential and maintain the health of broilers in order to gradually replace the AGPs (Yang et al., 2009; Bai et al., 2017). One such potential alternative of AGPs in poultry was *Bacillus subtilis* (*B. subtilis*), which could rebalance the intestinal microflora by creating an anaerobic environment and by competitively inhibiting the pathogen, improve growth performance by promoting intestinal development and by promoting the digestion and absorption functions, and enhance intestinal health by improving immune function (Guo et al., 2016; Gao et al., 2017; Jayaraman et al., 2017; Mahmouda et al., 2017). However, antibiotics can be withdrawn, but animals become more prone to infection, and it has negative effects on production yields (Gadde et al., 2017). The search for an approach to gradually replace antibiotics may serve as an effective way to reduce the use of AGPs and maintain the good performance and health of pullets at the same time. Rehman et al. (2012) demonstrated that the simultaneous administration of antibiotics and probiotics had the most significant positive effects on intestinal homeostasis by the stabilization of the intestinal microbial composition, which indicated that the probiotics were active when simultaneously applied with antibiotics and the simultaneous supplementation of antibiotics and probiotics was beneficial for the formation of salutary gut microbiota. Hence, the simultaneous supplementation of antibiotics and probiotics could serve as an approach to gradually replace antibiotics.

Pullets undergo fast physical development before laying period (0~16 weeks), which were the critical periods for organ development. According to the physiological characteristics and nutrient requirements of laying hens at different weeks, the periods before laying (0~16 weeks) could be divided into four stages: the developmental period of the digestive and immune systems (0~3 weeks), the developmental period of the gut microflora (4~6 weeks), the developmental period of the skeleton (7~12 weeks), and the developmental period of the reproductive organs (13~16 weeks) (Adi and Magray, 2012). Meanwhile, the interactions between the gut and commensal microbes have proved to play crucial roles in preventing pathogen colonization, enhancing hosts' metabolism of nutrient (Kohl, 2012; Yeoman et al., 2012), improving the digestion of indigestible polysaccharides (Montagne et al., 2003), and promoting the development of gastrointestinal mucosal immune system (Purchiaroni et al., 2013), which indicated that the alteration of the intestinal microbiota could be involved in the regulation of organ development at different periods. The caecal microbiota has the most abundant microbiota in the broilers' gut and could actually represent the whole gut microbiota of chickens (Torok et al., 2011; Polansky et al., 2016). The numbers of microbes reach 10^11^ per gram of caecal digesta during the first 3 days post hatching and start building and remain relatively stabilized for the following 30 days (Apajalahti and Graham, 2004; Adi and Magray, 2012), in which phase, nutrition is critical for the growth and development of the host and its intestinal microbiota. Considering that the AGPs would alter enteric epithelial homeostasis and enhance host susceptibility to pathogens (Willing et al., 2011), the caecal microbial membership could also be significantly altered (Costa et al., 2017). Overall, the substitution of antibiotics during these periods could influence the formation of intestinal microbiota, as well as the body maturation and sexual maturation, which could have further effects on the laying performance.

Hence, the simultaneous short-termed supplementation of AGP and *B. subtilis* at the early developmental stage of pullets, which combined the advantages of using antibiotics and probiotics, could be beneficial for the construction of salutary intestinal microbiota and the early growth and development of pullet. With the aim to reduce the use of AGPs and to maintain the growth of the pullets at the same time, the present study was performed to compare the effect of temporary periodic supplementation of AGPs and *B. subtilis* and the effect of supplementation of AGPs during the whole period (0~16 weeks) on the caecal microbiota composition, intestinal functions, and the growth and development of pullets from 0 to 16 weeks of ages.

## Materials and methods

### Ethics approval statement

This study was carried out in accordance with the recommendations established by the Ministry of Agriculture, P. R. China. All the birds and the experimental protocols in this study were approved by the Institution Animal Care and Use Committee of the Northwest A&F University (protocol number NWAFAC1008).

### Animals and experimental procedures

*B. subtilis* DSM17299 used in the present study was provided by Evonik Industries (Hanau, Germany), the dried powder of which was composed of 1.6 × 10^9^ CFU/g of *B. subtilis* in the spore form. Bacillus subtilis DSM17299 was approved as a feed additive in the European Union (Knap et al., 2011). Meanwhile, AGPs (Bacitracin Zinc and Colistin Sulfate) were bought from Wuhan Guangtu Technology Co., Ltd (China).

Based on a single factor completely randomized design, a total of 630 one-day-old Hy-Line Brown pullets were randomly assigned to five groups with seven repeats per treatment and 18 birds per repeat, including the following five treatments: (1) AGPs group: supplied with AGPs (a combination of bacitracin zinc 20 mg/kg and colistin sulfate 4 mg/kg, the same below) based on the corn soybean basal diet for all 16 weeks before laying; (2) BA3 group: supplied with AGPs and *B. subtilis* (DSM 17299, 500 mg/kg, the same below) based on basal diet for 0~3 weeks and then only supplied with basal diet for 4~16 weeks; (3) BA6 group: supplied with AGPs and *B.subtilis* based on basal diet for 0~6 weeks and then only supplied with basal diet for 7~16 weeks; (4) BA12 group: supplied with AGPs and *B.subtilis* based on basal diet for 0~12 weeks and then only supplied with basal diet for 13~16 weeks; (5) BA16 group: supplied with AGPs and *B.subtilis* based on basal diet for all 16 weeks. The basal diet was a standard diet commonly used in the northwestern part of China to meet standard recommendation (NY/T-2009) that fits the nutrient requirements at different stages. The composition and nutrient contents of the basal diet at four periods are included in Table 1. All chickens were kept in an environmentally controlled henhouse with double-floor metabolism cages. The brooding temperature, photoperiod, and feeds were made available according to the recommendation for Hy-Line Brown pullets, and water was available *ad libitum*.

**Table 1:** Composition and nutrient levels of basal diets (as-fed basis).

**Items**	**Content**			
	**0 to 3 weeks**	**4 to 6 weeks**	**7 to 12 weeks**	**13 to 16 weeks**
**INGREDIENTS (%)**				
Corn	42.91	61.00	63.67	63.97
Soybean meal	17.00	23.00	22.00	18.00
Extruded corn	20.00	–	–	–
Fermented soybean meal	6.00	4.00	–	–
Fish meal	3.00	2.00	1.00	–
Wheat bran	3.00	5.00	10.00	15.00
Chicken plasma gluten meal	2.00	–	–	–
Soybean oil	1.80	1.40	0.05	–
Glucose	1.50	–	0.30	–
CaHPO_4_	1.20	1.40	–	–
Limestone	1.00	1.20	1.30	1.30
NaCl	0.12	0.20	1.20	1.20
Lys	0.10	0.10	0.30	0.34
Choline chloride	0.10	0.06	–	–
*DL*-Met	0.05	0.06	–	–
Acidifier	0.04	0.40	0.02	0.03
Complex enzyme	0.03	0.03	–	–
Thr	0.02	0.02	0.03	0.03
Multi-minerals^a^	0.10	0.10	–	–
Multi-vitamin^b^	0.03	0.03	0.10	0.10
Total	100.00	100.00	0.03	0.03
**NUTRIENT LEVELS (%)**				
CP	20.00	19.70	17.50	16.00
Ca	0.82	0.91	0.83	0.79
Total P	0.62	0.63	0.61	0.61
Available phosphorus	0.38	0.40	0.36	0.35
NaCl	0.37	0.34	0.37	0.37
Lys	1.18	1.11	0.87	0.75
Met	0.39	0.39	0.31	0.29
Met+Cys	0.75	0.72	0.63	0.59

a *The trace element premix provided per kg of diet: Cu (as copper sulfate) 10 mg, Fe (as ferrous sulfate) 80 mg, Mn (as manganese sulfate) 80 mg, Zn (as zinc sulfate) 75 mg, I (as potassium iodide) 0.40 mg, Se (as sodium selenite) 0.30 mg*.

b *The vitamin premix provided per kg of diet: vitamin A, 250,000 IU; vitamin D, 50,000 IU; vitamin K3, 53 mg; vitamin B1, 40 mg; vitamin B2, 120 mg; vitamin B12, 0.50 mg; vitamin E, 600 IU; biotin, 0.65 mg; folic acid, 25 mg; pantothenic acid, 240 mg; niacin, 1,000 mg*.

### Growth performance

The experiment lasted for 16 weeks. At 3, 6, 12, and 16 weeks of age, chickens were weighed by pen (replication), and the feed consumption was recorded by replication. Average body weight (ABW), average daily gain (ADG), average daily feed intake (ADFI), and feed conversion ratio (FCR, also known as the ratio of feed to gain) were calculated for periods of 0~3, 4~6, 7~12, and 13~16 weeks.

### Sample collection and determination of characteristic growth indexes

At 3, 6, 12, and 16 weeks of age in the trial, one bird per replicate was selected (based on the average weight of each replicate), weighed, and killed by cervical dislocation. For each sampling, by removing the contamination of intestinal contents, the complete middle segments of duodenum, jejunum, and ileum of the length 3 cm were collected and fixed in 10% buffered formalin for at least 48 h for further histological processing. Meanwhile, the duodenal, jejunal, and ileal mucosa samples, as well as the duodenal, jejunal, ileal, and caecal content samples were collected in 2 mL Eppendorf tubes and frozen immediately in liquid nitrogen.

Later, different characteristic growth indexes were specifically measured at different weeks of age. At 3 and 6 weeks of age, the immune organs (thymus, spleen, and bursa) and the digestive organs (crop, glandular, gizzard, duodenum, jejunum, ileum, and caecum) were removed and weighed immediately. Organ indexes were expressed relative to the body weight (g of organ/kg of BW). At 12 weeks of age, the tibia and keel were collected by picking the muscle from the bones and then were used to measure the length of the bones (cm). At 16 weeks of age, abdominal fat was separated from subcutaneous tissues and then weighed (g).

### Intestinal morphology

The complete middle segments of duodenum, jejunum, and ileum (3 cm), which were fixed in 10% buffered formalin, were used for the analysis of intestinal morphology. After fixing, the samples were dehydrated and cleared. Subsequently, the intestinal samples were cut and inserted into cassettes, which were embedded in liquid paraffin. Later, a 5 μm paraffin section was obtained using a microtome and was stained with hematoxylin-eosin (Liu et al., 2017b). Villus height and crypt depth were determined using a phase contrast microscope (Naghi et al., 2016), and the villus height to crypt depth ratio (VH/CD) was calculated.

### Measurement of activities of small intestinal amylase and contents of secreted immunoglobulin a (sIgA)

The duodenal and jejunal mucosa were homogenized together with normal saline (1:4) using a homogenizer. Later, the mucosal homogenates were centrifuged at 3,000 × g for 15 min at 4°C. Supernatants were collected to determine sIgA levels according to the manufacturer's instructions provided with the commercial kit (China Institute of Atomic Energy, Beijing, China). The protein concentration was detected by the Coomassie Brilliant Blue method, and the sIgA content was expressed as μg/100 mg protein.

At 3 and 6 weeks of age, the activity of small intestinal amylase was measured using the α-Amylase Assay Kit (Nanjing Jiancheng Bioengineering Institude, Nanjing, China). Briefly, before the analysis of amylase, intestinal content was defrosted and diluted with normal saline to 1:10 and was homogenized together with normal saline using homogenizer. Subsequently, the content of the homogenate was centrifuged at 3,500 × g for 10 min at 4°C, and the supernatant was collected to determine amylase activity according to the manufacturer's instructions (Nanjing Jiancheng Bioengineering Institude, Nanjing, China). The protein concentrations in all samples were determined using the Coomassie Brilliant Blue method (Sedmak and Grossberg, 1977). The amylase activity was expressed as U/mg protein.

### The relative mRNA expression of sucrase in the duodenal, jejunal, and ileal mucosa

Total RNA and cDNA of mucosal samples were obtained using TRIzol and the Primer Script RT Reagent Kit (TaKaRa, Dalian, China). Total RNA was stored at −80°C, and cDNA was stored at −20°C until use. The mRNA expression level of sucrose was quantified by quantitative real-time PCR (qPCR). The assay was carried out using the SYBR Premix Ex Taq Kit (TaKaRa, Dalian, China) on IQ5 (Bio-Rad, Hercules, USA). The PCR conditions used were 95°C for 5 min; 40 cycles of 95°C for 10 s, 60°C for 30 s, and 72°C for 30 s; followed by 72°C for 5 min. The sucrase gene was amplified using primers (F: CGCAAAAGCACAGGGACAGT, R: TCGATACGTGGTGTGTGCTCAGTT) as described by Speier et al. (2012). All samples were run in triplicates, and the average cycle threshold (Ct) values were calculated for quantification using the 2^−ΔΔCt^ method (Livak and Schmittgen, 2001).

#### Caecum microbiota 16S rRNA sequencing analysis

At 3, 6, 12, and 16 weeks of age, five samples of caecal contents obtained separately from five random replicates were used to extract DNA for subsequent 16S rRNA sequencing analysis and absolute qPCR.

The DNA extraction and 16S rRNA sequencing of a total of 70 samples were performed at Shanghai Personal Biotechnology Co., Ltd. In brief, DNA was extracted using the QIAamp DNA Stool Mini Kit (QIAGEN, Dusseldorf, Germany) and was amplified using the 520F/802R primer set (520F: 5′-AYTGGGYDTAAAGNG-3′, 802R: 5′-TACNVGGGTATCTAATCC-3′), which targets the V4 region of the bacterial 16S rRNA (Pyrobest DNA Polymerase, TaKaRa, DR500A). The PCR products were purified using the AxyPrep DNA Gel Extraction Kit (Axygen, America). Pyrosequencing was conducted on an Illumina MiSeq platform, and library construction was performed using the paired-end sequencing method. Detailed analysis was performed in accordance with a previous study (Ding et al., 2017).

#### Determination of bacterial number in caecum by absolute qPCR

The metagenomic DNA of the caecum content was extracted using the modified cetyltrimethyl ammonium bromide (CTAB) method (Minas et al., 2011). The quantity and purity of total DNA were analyzed using a NanoDrop® ND-2000 spectrophotometer (Thermo Scientific, MA, USA), and the integrity and quality of DNA were analyzed by using agarose gel (1%) electrophoresis. Afterwards, 5 μL of the DNA sample was diluted to 30 ng/μL and then stored at −20°C. Meanwhile, all the dilution ratios were recorded.

The populations of *Clostridium leptum* (*C*. *leptum*), *Bacteroides fragilis* (*B*. *fragilis*), *Escherichia coli* (*E*. *coli*), and *Salmonella enterica* (*S*. *enterica*) in the caecum content were determined by SYBR green-based absolute qPCR. The bacteria specific primer sets were shown in Table 2. The qPCR plasmid standard was prepared according to the method described in a previous study (Liu et al., 2017a). After that, the concentration of plasmid standards of *C*. *leptum, B*. *fragilis, E*. *coli*, and *S*. *enterica* was diluted to 20 ng/μL and then subjected to a series of tenfold dilutions (10^−1^~10^−6^) to obtain the standard curve. The copy number of the diluted plasmid standard was calculated as follows: copy number/μL = [N_A_ × C(ng/μL) × 10^−9^]/[660(Dalton/bp) × L_DNA_(bp)], where N_A_ = 6.02 × 10^23^; C (ng/μL) = plasmid DNA concentration (20 ng/μL); L_DNA_(bp) = lengh of plasmid DNA (pMD-19T vector + target fragment). The standard curve of the bacteria was calculated based on the copy number and Ct values. Standard curve and equation are shown in Supplementary Figure 1.

**Table 2:** Primers for qPCR analysis by 16S rRNA.

**Bacteria**	**Primer sequences (5′to 3′)**	**Product size (bp)**	**References**
*C. leptum*	F:GCACAAGCAGTGGAGT	239	Matsuki et al., 2004
	R:CTTCCTCCGTTTTGTCAA		
*B. fragilis*	F:CACTTGACTGTTGTAGATAAAG	135	Papaparaskevas et al., 2003
	R:CATCTTCATTGCAGCATTATCC		
*E. coli*	F:CATGCCGCGTGTATGAAGAA	96	Huijsdens et al., 2002
	R:CGGGTAACGTCAATGAGCAAA		
*S. enteritidi*s	F:GGAGTCTTGTAGAGGGGGG	334	Self-designed
	R:AGGTAAGGTTCTTCGCGTTG		

All the diluted samples were amplified by qPCR using the conditions mentioned above, and the reaction mixture (20 μL) consisted of 10 μL of SYBR Premix Ex Taq (TaKaRa, Dalian, China), 1 μL of forward primer, 1 μL of reverse primer, 7 μL of sterile deionized water, and 1 μL of DNA (30 ng). The number of *C*. *leptum, B*. *fragilis, E*. *coli*, and *S*. *enterica* in each sample with 30 ng metagenomic DNA could be calculated by the following equation: X = lg[(M_DNA_/M_C_) × (C30/30) × D], where M_DNA_ represents the weight of total DNA in the sample (ng); M_C_ represents the weight of the content we used (g); C30 represents the copy number of 30 ng metagenomic DNA (plug Ct values into the standard curve); and D represents the dilution ratio. Specifically, the copy numbers of each reaction were calculated from the standard curves, and the populations of bacteria were expressed as log_10_ CFU/g content.

## Statistical analysis

For all indexes except for the 16s rRNA gene sequencing, the analysis was done by one-way ANOVA using the SPSS 21.0 software with replicates as experimental units, and the differences were considered to be statistically significant at *P* < 0.05. The significant differences at the 0.05 level due to the treatments were separated by Duncan's multiple range tests. Furthermore, the correlations between caecal microbiota and intestinal morphology from 0 to 16 weeks were analyzed using the Pearson correlation analysis procedure of SPSS 21.0, and *P* < 0.05 was considered as a significant correlation.

## Results

### Growth performance

No differences (*P* > 0.05) were observed in growth performance among treatments at any period of time (Table 3). Specifically, simultaneous supplementation of AGP and *B. subtilis* for a short period (0~3 weeks) could maintain normal growth in 0~16-week-old pullets, which was similar to the effect of supplementation with AGPs for the whole period (0~16 weeks).

**Table 3:** Growth performance in pullets fed on different dietary treatments from 0 to 16 weeks.

**Parameters**	**AGP**	**BA3**	**BA6**	**BA12**	**BA16**	**SEM**	***P-*value**
**AVERAGE BODY Weight (g/BIRD)**							
3 week	182.62	177.64	176.70	178.26	176.81	0.91	0.215
6 week	481.09	461.99	477.84	472.71	467.89	2.60	0.133
12 week	971.16	978.25	926.86	952.84	958.07	9.56	0.506
16 week	1321.66	1326.35	1280.76	1322.31	1306.17	8.13	0.384
**AVERAGE FEED INTAKE (g/BIRD/DAY)**							
3 week	17.56	17.29	17.13	17.67	17.23	0.09	0.323
6 week	46.23	46.58	46.16	50.78	51.85	1.55	0.659
12 week	63.37	60.70	57.82	59.46	56.86	1.25	0.521
16 week	84.08	85.41	81.93	85.18	84.63	0.51	0.192
**AVERAGE DAILY WEIGHT GAIN (g/BIRD/DAY)**							
3 week	7.11	6.86	6.79	6.88	6.80	0.04	0.134
6 week	13.65	12.87	13.38	13.78	13.40	0.15	0.386
12 week	11.74	11.71	10.62	11.41	11.07	0.24	0.548
16 week	9.86	9.58	9.45	9.60	9.53	0.18	0.972
**FEED CONVERSION RATIO (FEED/GAIN)**							
3 week	2.47	2.52	2.53	2.57	2.53	0.02	0.642
6 week	3.39	3.64	3.47	3.69	3.85	0.11	0.711
12 week	5.41	5.18	5.45	5.24	5.15	0.06	0.304
16 week	8.73	8.98	8.78	9.05	8.89	0.19	0.983

*AGP, the AGPs group (supplemented with AGPs based on basal diets for 16 weeks); BA3, the BA3 group (supplemented with AGPs and B. subtilis based on basal diets for 3 weeks); BA6, the BA6 group (supplemented with AGPs and B. subtilis based on basal diets for 6 weeks); BA12, the BA12 group (supplemented with AGPs and B. subtilis based on basal diets for 12 weeks); BA16, the BA16 group (supplemented with AGPs and B. subtilis based on basal diets for 16 weeks)*.

### Characteristic growth indexes at different periods

Characteristic growth indexes at different periods are shown in Table 4. At 3 weeks of age, only the activity of ileac amylase in simultaneous supplementation groups improved when compared with the AGP group (*P* = 0.028). No differences (*P* > 0.05) occurred in characteristic growth indexes at 6, 12, and 16 weeks of ages among treatments, which suggested that the simultaneous supplementation of AGPs and *B. subtilis* could maintain growth and development when compared with the AGPs group.

**Table 4:** Immune and digestive performance in 0~6-week-old pullets and skeleton growth and abdominal fat deposition in 7~16-week-old pullets.

**Items**		**AGP**	**BA3**	**BA6**	**BA12**	**BA16**	**SEM**	***P-*value**
**IMMUNE AND DIGESTIVE PERFORMANCE AT 3 WEEKS OF AGE**								
Organ indexes (g/kg)	Thymus	6.00	5.77	5.97	5.80	5.86	0.17	0.991
	Spleen	1.78	1.67	2.13	1.58	2.21	0.53	0.372
	Bursa of fabricius	5.84	5.47	6.07	6.01	5.68	0.18	0.783
	Crop	5.15	4.66	4.63	4.61	5.05	0.11	0.647
	Proventriculus	7.31	7.44	7.91	7.39	7.74	0.10	0.664
	Gizzard	37.82	40.55	38.24	39.65	39.94	0.16	0.611
	Duodenum	12.70	12.09	14.06	11.07	11.44	0.39	0.119
	Jejunum	17.72	17.50	18.26	17.23	17.78	0.34	0.917
	Ileum	14.73	11.86	13.32	13.16	13.16	0.35	0.143
	Caecum	6.29	6.03	6.58	6.18	7.06	0.18	0.395
Amylase activity (U/mgprot)	Duodenum	9.19	28.73	–	–	–	8.61	0.084
	Jejunum	92.99	65.08	–	–	–	46.37	0.564
	Ileum	129.87^b^	197.60^a^	–	–	–	25.27	0.028
**IMMUNE AND DIGESTIVE PERFORMANCE AT 6-WEEKS OF AGE**								
Weight homogeneity (%)	79.53	86.03	83.45	–	–	2.20	0.542
Organ indexes (g/kg)	Thymus	5.15	5.77	5.33	5.23	4.84	0.20	0.711
	Spleen	4.06	3.00	3.19	3.37	3.83	0.19	0.083
	Bursa of Fabricius	1.39	1.39	1.14	1.18	1.17	0.28	0.342
	Crop	4.33	4.55	5.42	4.68	4.39	0.22	0.151
	Proventriculus	6.36	6.05	6.73	5.69	6.45	0.17	0.198
	Gizzard	36.30	34.44	37.39	32.96	34.38	0.49	0.492
	Duodenum	9.69	10.22	10.44	8.78	9.34	0.26	0.268
	Jejunum	15.20	14.83	16.54	14.09	14.58	0.32	0.147
	Ileum	10.72	10.46	11.70	10.32	10.50	0.23	0.343
	Caecum	6.43	6.07	6.87	5.67	6.50	0.16	0.151
Amylase activity (U/mgprot)	Duodenum	4.32	8.37	11.36	–	–	1.92	0.348
	Jejunum	284.20	613.60	347.09	–	–	75.70	0.172
	Ileum	194.17	232.83	269.21	–	–	29.99	0.645
**LENGTH OF TIBIA AND KEEL AT 12 WEEKS OF AGE**								
Length of tibia (cm)		10.13	9.80	9.76	9.99	–	0.06	0.127
Length of and keel (cm)		9.40	9.34	9.32	9.36	–	0.02	0.410
**ABDOMINAL FAT WEIGHT AT 16 WEEKS OF AGE**								
Abdominal fat (g)		5.85	3.75	4.22	4.50	7.74	0.54	0.116

*AGP, the AGPs group (supplemented with AGPs based on basal diets for 16 weeks); BA3, the BA3 group (supplemented with AGPs and B. subtilis based on basal diets for 3 weeks); BA6, the BA6 group (supplemented with AGPs and B. subtilis based on basal diets for 6 weeks); BA12, the BA12 group (supplemented with AGPs and B. subtilis based on basal diets for 12 weeks); BA16, the BA16 group (supplemented with AGPs and B. subtilis based on basal diets for 16 weeks). The different superscripted capital letters in the same segment indicate an adjusted significant difference at P < 0.05*.

### Intestinal morphology

The effect of simultaneous phased supplementation of *B. subtilis* and AGPs on the villus height, crypt depth, and the ratio of villus height/crypt depth (VH/CD) in small intestine are shown in Table 5. At 3 weeks of age, when compared with the AGPs group, the crypt depth in duodenum decreased (*P* = 0.001) in the BA3 group. A decreased villus height (*P* = 0.049) and crypt depth (*P* = 0.001) in jejunum were observed in BA3 groups. At 12 weeks of age, the villus height of jejunum increased (*P* = 0.046) in the BA3 group when compared with the BA6 and AGP groups. In the ileum, when compared with the AGPs group, all simultaneous supplementation groups increased the villus height and the ratio of VH/CD and decreased the crypt depth (*P* = 0.002). At 16 weeks of age, an increased (*P* = 0.038) villus height in jejunum was determined in the BA16 group when compared with the BA6 and AGP groups.

**Table 5:** Villus height, crypt depth, and villus height/crypt depth ratio in the small intestine of pullets fed on different dietary treatments from 0 to 16 weeks.

**Parameters**	**Period (weeks)**	**AGP**	**BA3**	**BA6**	**BA12**	**BA16**	**SEM**	***P-*value**
**DUODENUM**								
Villus height (VH) (μm)	3	480.98	464.95	–	–	–	36.43	0.668
	6	665.27	592.60	602.51	–	–	14.65	0.083
	12	600.57	596.93	612.52	628.60	–	12.22	0.245
	16	619.46	586.26	639.50	608.88	608.05	10.38	0.579
Crypt depth (CD) (μm)	3	56.35^a^	44.40^b^	–	–	–	2.71	0.001
	6	68.86	68.53	69.59	–	–	1.54	0.964
	12	49.44	49.38	46.83	49.34	–	0.95	0.817
	16	53.79	54.31	53.17	56.21	53.22	0.74	0.729
VH/CD	3	8.59	10.62	–	–	–	0.96	0.058
	6	9.66	8.74	8.81	–	–	0.28	0.338
	12	12.22	12.11	13.05	13.07	–	0.34	0.618
	16	11.58	10.82	12.01	10.89	11.44	0.20	0.265
**JEJUNUM**								
Villus height (VH) (μm)	3	263.04^a^	234.86^b^	–	–	–	12.60	0.049
	6	311.89	346.20	342.39	–	–	11.41	0.134
	12	364.29^b^	421.54^a^	351.80^b^	377.32^ab^	–	9.63	0.046
	16	385.24^b^	435.25^ab^	399.59^b^	422.24^ab^	494.85^a^	12.20	0.038
Crypt depth (CD) (μm)	3	43.43^a^	36.70^b^	–	–	–	1.47	0.001
	6	48.29	49.49	49.91	–	–	1.00	0.562
	12	49.31	53.98	46.21	51.00	–	1.11	0.085
	16	50.13	53.05	54.04	55.53	53.35	0.76	0.203
VH/CD	3	6.08	6.44	–	–	–	0.43	0.423
	6	6.49	7.00	6.89	–	–	0.21	0.140
	12	6.91	7.60	6.91	7.34	–	0.16	0.347
	16	7.70	8.24	7.41	7.60	9.34	0.24	0.065
**ILEUM**								
Villus height (VH) (μm)	3	249.41^a^	220.51^b^	–	–	–	12.45	0.039
	6	281.34	252.94	279.54	–	–	8.04	0.158
	12	290.41^b^	373.96^a^	358.58^a^	371.01^a^	–	11.97	0.023
	16	405.97	370.11	380.81	380.75	440.07	12.71	0.460
Crypt depth (CD) (μm)	3	42.51	40.43	–	–	–	3.78	0.593
	6	45.26	45.24	45.84	–	–	1.08	0.509
	12	51.20^a^	46.80^b^	42.66^b^	43.51^b^	–	1.01	0.002
	16	49.29	51.75	52.27	51.62	51.52	0.74	0.786
VH/CD	3	5.97	5.55	–	–	–	0.47	0.386
	6	6.28	5.61	6.09	–	–	0.16	0.073
	12	6.30^b^	7.70^a^	8.27^a^	8.12^a^	–	0.25	0.012
	16	8.24	7.18	7.25	7.42	8.63	0.25	0.260

*AGP, the AGPs group (supplemented with AGPs based on basal diets for 16 weeks); BA3, the BA3 group (supplemented with AGPs and B. subtilis based on basal diets for 3 weeks); BA6, the BA6 group (supplemented with AGPs and B. subtilis based on basal diets for 6 weeks); BA12, the BA12 group (supplemented with AGPs and B. subtilis based on basal diets for 12 weeks); BA16, the BA16 group (supplemented with AGPs and B. subtilis based on basal diets for 16 weeks). The different superscripted capital letters in the same segment indicate an adjusted significant difference at P < 0.05*.

### Expression levels of intestinal brush border membrane enzyme activities of sucrase

**Figure 1:**
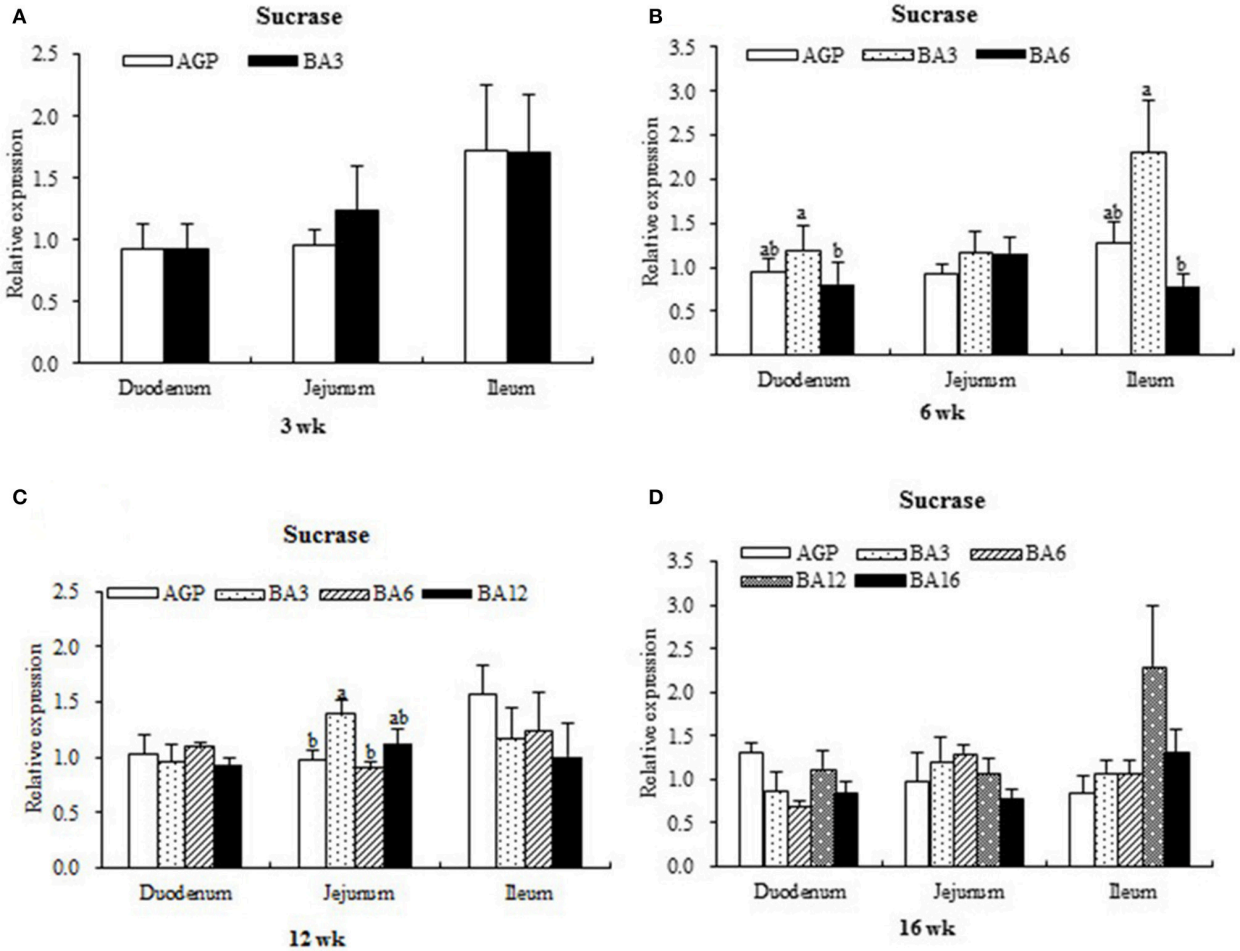
Transcript level of sucrase in the intestinal mucosa of pullets in 0~16 weeks. The intestinal positions include duodenum, jejunum, and ileum. **(A)** Relative expression of sucrase at week 3. **(B)** Relative expression of sucrase at week 6. **(C)** Relative expression of sucrase at week 12. **(D)** Relative expression of sucrase at week 16.

The expression levels of sucrase in intestinal mucosa at each period are presented in Figure 1. At week 6, when compared with the pullets in the BA6 group, the concentrations of sucrase in the mucosa of duodenum (*P* = 0.021) and ileum (*P* = 0.046) were found to be elevated in the BA3 group. At week 12, jejunal sucrase expression increased (*P* = 0.035) in the BA3 group when compared with the AGP and BA6 groups. However, neither the AGPs nor the simultaneous supplementation of AGPs and *B. subtilis* altered sucrase expression (*P* > 0.05) in the intestine at weeks 3 and 16.

### sIgA content in intestinal mucosa

In duodenum, the contents of sIgA in mucosa were not affected (*P* > 0.05) by the dietary supplementation of AGP or the simultaneous supplementation of AGPs and *B. subtilis* (Table 6). In jejunum, the sIgA contents in mucosa of the BA3 group increased (*P* < 0.001) when compared with the AGP and BA6 groups at week 16 (Table 6).

**Table 6:** The sIgA content in the duodenum and jejunum mucosa of pullets fed on different dietary treatments from 0 to 16 weeks (ng/100 mg protein).

**Parameters**	**AGP**	**BA3**	**BA6**	**BA12**	**BA16**	**SEM**	***P-*vaule**
**IN DUODENUM**							
3 week	8.55	8.03	–	–	–	2.52	0.841
6 week	7.49	7.50	8.01	–	–	0.39	0.848
12 week	5.55	5.63	5.71	5.83	–	0.10	0.828
16 week	6.55	7.53	6.85	7.59	7.05	0.17	0.250
**IN JEJUNUM**							
3 week	1.79	1.00	–	–	–	0.61	0.227
6 week	3.33	4.14	3.00	–	–	0.32	0.366
12 week	27.74	31.03	30.09	33.59	–	1.07	0.273
16 week	10.49^b^	12.73^a^	8.25^c^	12.12^ab^	11.66^ab^	0.40	<0.001

*AGP, the AGPs group (supplemented with AGPs based on basal diets for 16 weeks); BA3, the BA3 group (supplemented with AGPs and B. subtilis based on basal diets for 3 weeks); BA6, the BA6 group (supplemented with AGPs and B. subtilis based on basal diets for 6 weeks); BA12, the BA12 group (supplemented with AGPs and B. subtilis based on basal diets for 12 weeks); BA16, the BA16 group (supplemented with AGPs and B. subtilis based on basal diets for 16 weeks). The different superscripted capital letters in the same segment indicate an adjusted significant difference at P < 0.05*.

### Similarities between the microbial communities

**Figure 2:**
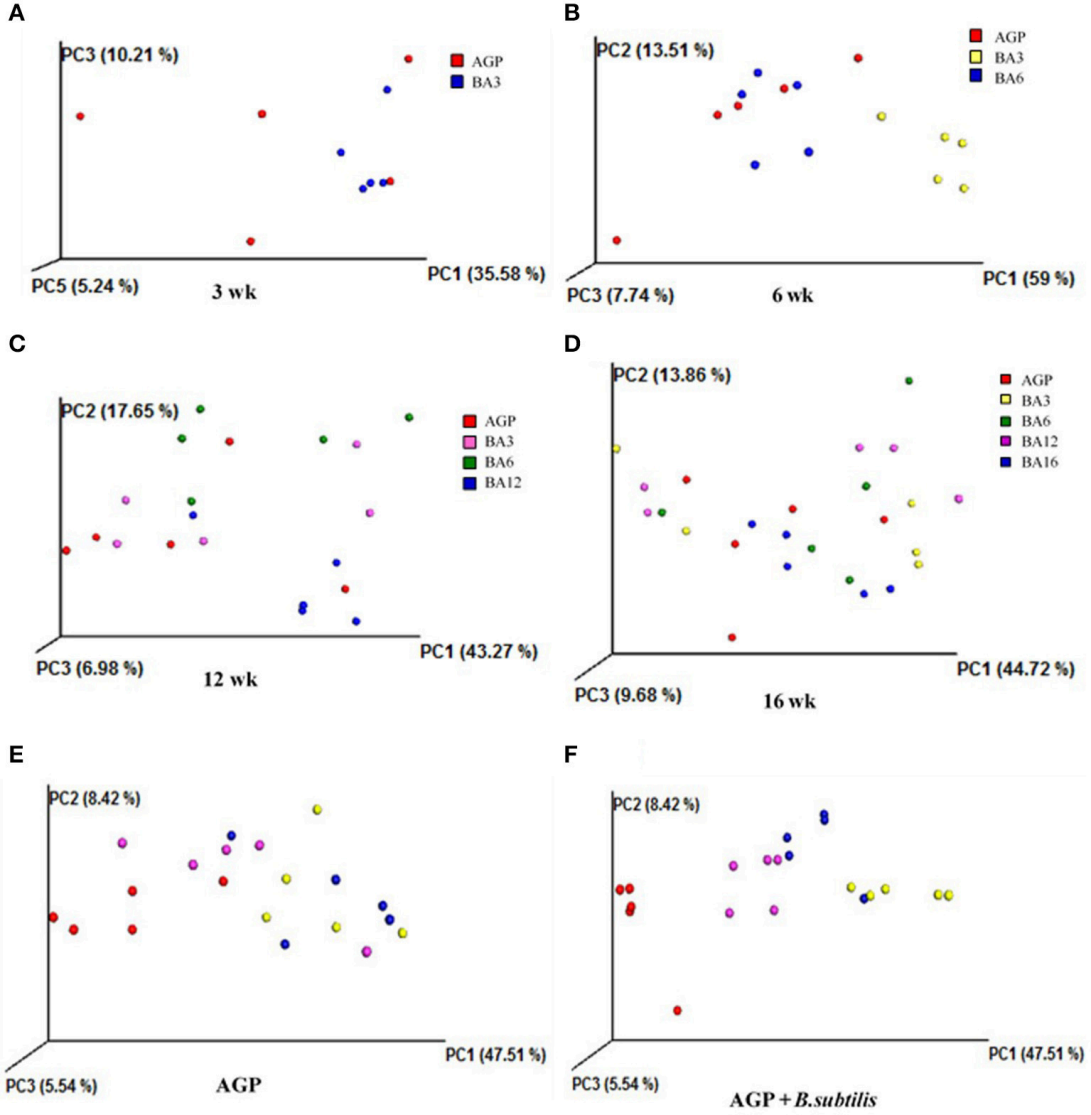
Principal component analysis (PCoA) plots based on treatments of pullets from 0 to 16 week-age. The plots represent the samples, and the different colors represent treatments. **(A)** PCoA plots for treatments at week 3; **(B)** PCoA plots for treatments at week 6; **(C)** PCoA plots for treatments at week 12; **(D)** PCoA plots for treatments at week 16; **(E)** PCoA plots in AGP group for bird ages; **(F)** PCoA plot in AGP + *B. subtilis* group for bird ages.

The weighted principal component analysis (PCoA) UniFrac beta diversity plots were obtained in the present study (Figure 2). At weeks 3 and 6, the samples in the BA3 and BA6 groups were separately clustered, while the samples in AGP group were badly clustered (Figures 2A,B). However, the samples failed to reveal any clustering patterns based on different treatments at weeks 12 and 16 (Figures 2C,D).

We also clustered the samples from the AGP and BA16 groups separately by periods. In terms of AGP treatments, the PCoA plots failed to exhibit any clustering patterns by periods (Figure 2E); in contrast, PCoA plots of simultaneous supplementation group exhibited evident clustering by periods (Figure 2F). Overall, our results indicated that the simultaneous phased supplementation of AGPs and *B. subtilis* could maintain the stability of the intestinal flora, which was beneficial for the intestinal health and function, whereas the chronic supplementation of AGPs could not maintain the stability of the intestinal microbiota.

### Predominant microbes and significantly altered caecal microbiota

**Figure 3:**
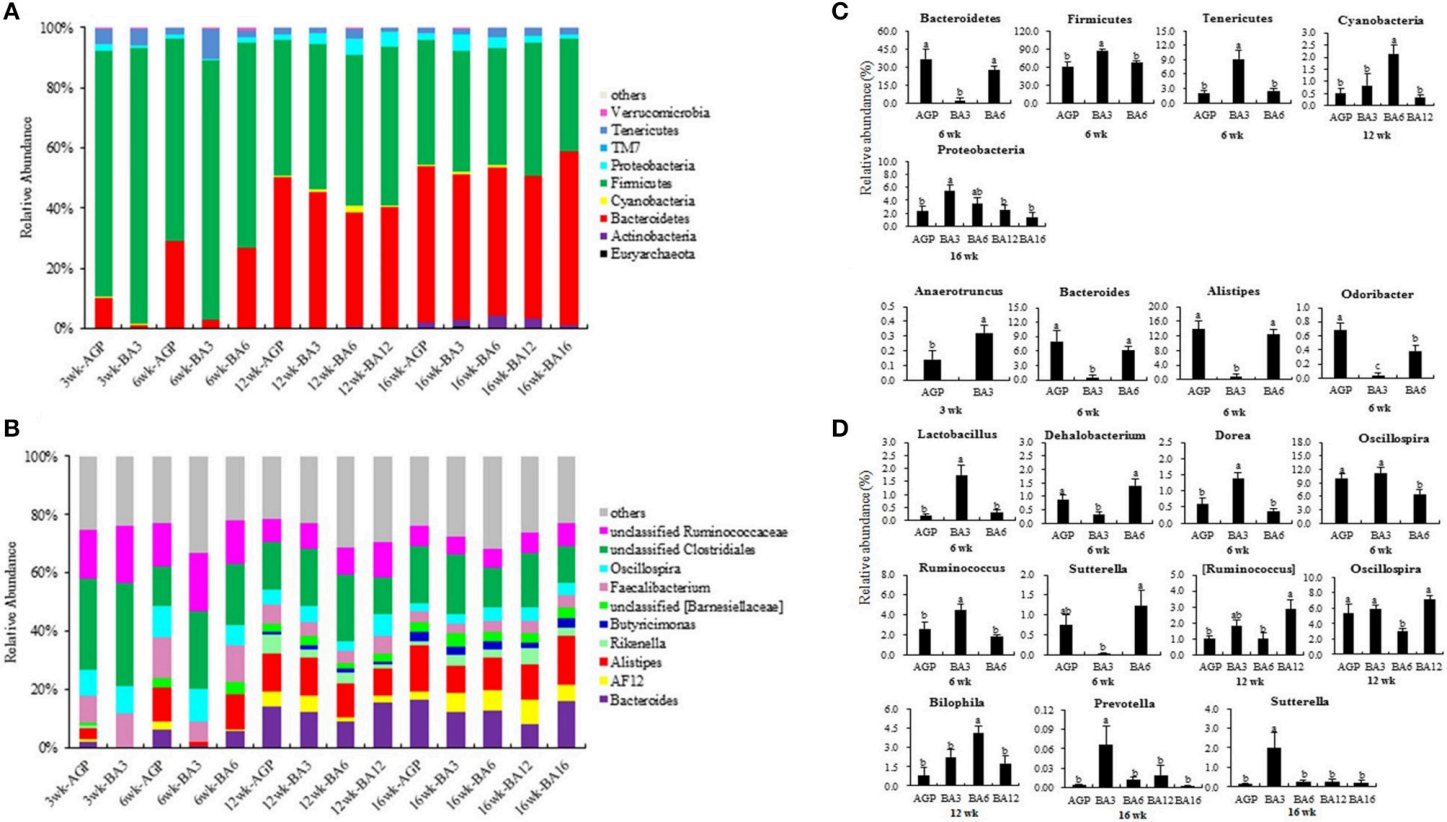
Composition of the caecal microbiome of pullets from different groups. The caecal microbiota at phylum **(A)** and genus **(B)** levels, the differentially abundant phylum **(C)** and genera **(D)** in caecum of pullets from 0 to 16 weeks (*P* < 0.05). For **(A)** and **(B)**, a color-coded bar plot shows the average bacterial phylum and genus distribution in different mixed groups and AGP group from 0 to 16 weeks.

The most dominant microbiota at phylum and genus levels is shown in Figures 3A,B. By using PICRUSt, the roles of microbial communities in caecum in the metabolism of amino acid, carbohydrate, and energy and the trans-membrane transport of nutrients were all verified (Supplementary Figures 2–5). Here, the significantly altered microbiota was further identified. As shown in Figures 3A,C, the BA3 group has greater relative abundance of *Firmicute* (*P* = 0.008) and *Tenericutes* (*P* = 0.002) and lower relative abundance of *Bacteroidetes* (*P* = 0.001) when compared with the BA6 and AGP groups at 6 weeks of age. At 12 weeks of age, the relative abundance of *Cyanobacteria* in the BA6 group was increased (*P* = 0.004) when compared with the other groups. At 16 weeks of age, the relative abundance of *Proteobacteria* in the BA3 group was greater (*P* = 0.025) than the other groups.

At genus level (Figures 3B,D), *Anaerotruncus* was the only altered microbe at 3 weeks of age, which increased in the BA3 group when compared with the AGP group (*P* = 0.049). At 6 weeks of age, the abundances of *Bacteroides* (*P* = 0.006), *Alistipes* (*P* < 0.001), and *Odoribacter* (*P* < 0.001), which belonged to phylum *Bacteroidetes*, decreased in the BA3 group when compared with the AGP and BA6 groups (Figures 3B,D). In contrast, within phylum *Firmicutes*, the abundances of *Lactobacillus* (*P* = 0.001), *Dorea* (*P* = 0.002), and *Ruminococcus* (*P* = 0.007) were all increased in the BA3 group when compared with the AGP and BA6 groups. Abundance of the genus *Dehalobacterium* (*P* = 0.004) and genus *Sutterella* (*P* = 0.029) in the BA3 group decreased when compared with the AGP and BA6 groups. Moreover, the BA3 and AGP groups had a greater abundance of *Oscillospira* (*P* = 0.023) when compared with the BA6 group. At 12 weeks of age, the abundance of *Ruminococcus* in the BA12 group was higher (*P* = 0.016) than the AGP and BA6 groups; in addition, more *Bilophila* (*P* = 0.009) and less *Oscillospira* (*P* = 0.006) in the BA6 group was identified when compared with the other groups. Whereas, at 16 weeks of age, the abundances of *Prevotella* (*P* = 0.046) and *Sutterella* (*P* = 0.006) in the BA3 group were greater than those of the other groups.

### Microbial populations in caecum detected by absolute qPCR

**Figure 4:**
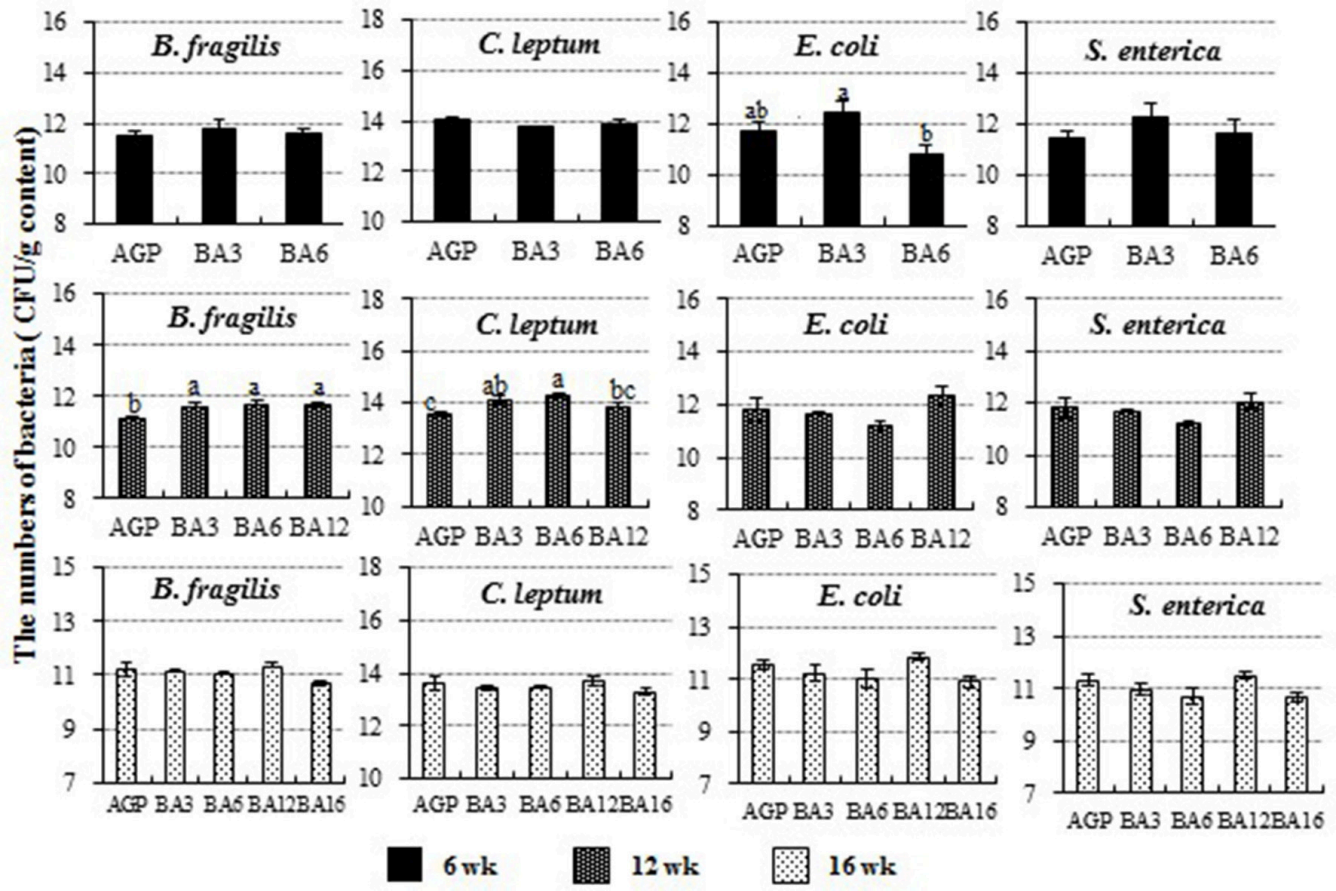
The population of *Clostridium leptum, Bacteroides fragilis, Escherichia coli*, and *Salmonella enterica* in caecum of pullets from 0 to 16 weeks. The bars represent the numbers of the bacteria in caecum per g content, and different patterns represent different ages.

The populations of *C*. *leptum, B*. *fragilis, E*. *coli*, and *S*. *enterica* in caecum content detected by absolute qPCR are shown in Figure 4. At 6 weeks of age, the number of *E. coli* species (*Proteobacteria, Enterobacteriaceae, Escherichia*) in the BA3 group was greater than the BA6 group (*P* = 0.042). At 12 weeks of age, the populations of *B. fragilis* species (*Bacteroidetes, Bacteroidaceae, Bacteroides*) in the AGP group was decreased when compared with the simultaneous supplementation groups (*P* = 0.011). Meanwhile, the population of *C. leptum* (*Firmicutes, Clostridia* IX cluster, including species in genus *Ruminococcus, Eubacterium, Clostridium*, and *Faecalibacterium*) was increased in the BA3 and BA6 groups when compared with the AGP group (*P* = 0.004). However, no changes were observed at 16 weeks of age (*P* > 0.05).

### Pearson correlation between caecal microbiota and intestinal morphology

**Figure 5:**
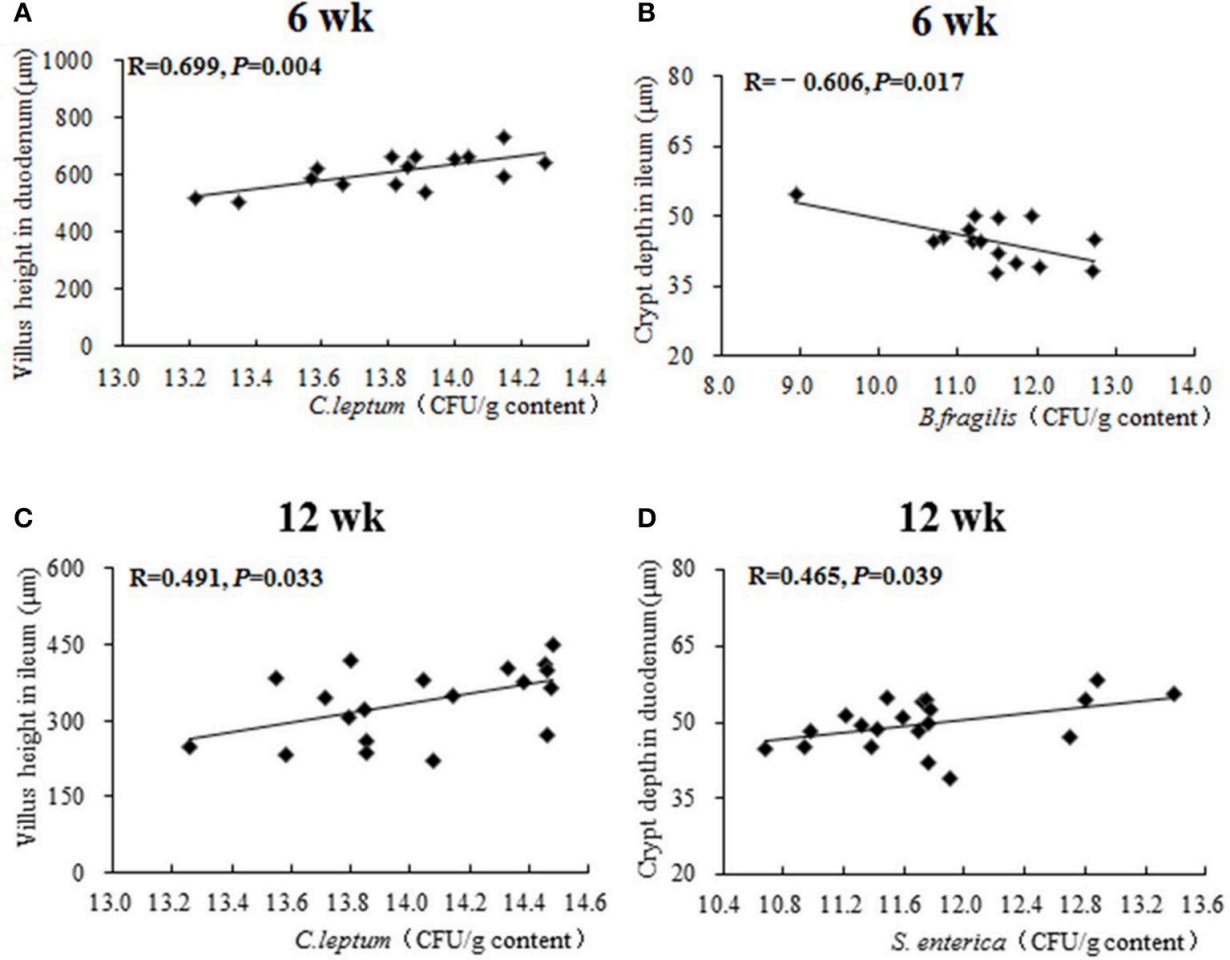
The Pearson correlation analysis between caecal microbiota and intestinal morphology. The plots represent the samples in all treatments from 0 to 16 weeks, the transverse line represents the linear relationship between caecal microbiota and intestinal morphology. *R-*value: Pearson correlation coefficient, *R* > 0 represents positive correlation: *R* < 0 represents negative correlation. *P-*value: significance was declared at *P* < 0.05. **(A)** Correlational relationship between population of caecal *C. leptum* and villus height in duodenum at week 6; **(B)** Correlational relationship between population of caecal *B. fragilis* and crypt depth in ileum at week 6; **(C)** Correlational relationship between population of caecal *C. leptum* and villus height in ileum at week 12; **(D)** Correlational relationship between population of caecal *S. enterica* and crypt depth in duodenum at week 12.

Results of Pearson correlation analysis between caecal microbiota and intestinal morphology are shown in Figure 5. At 6 weeks of age, both a positive correlation between the population of caecal *C. leptum* and duodenal villus height (*R* = 0.699, *P* = 0.004) and a negative correlation between the population of caecal *B. fragilis* and ileal crypt depth (*R* = −0.606, *P* = 0.017) were identified. At 12 weeks of age, a positive correlation between population of caecal *C. leptum* and ileal villus height (*R* = 0.491, *P* = 0.033) and a positive correlation between population of caecal *S. enterica* and duodenal crypt depth (*R* = 0.699, *P* = 0.004) were also identified.

## Discussion

The AGPs exerted antimicrobial effects when they entered the gastrointestinal tract as the pullets consumed the diet post hatching, which was beneficial for the maintenance of health and productivity but was harmful for the maintenance of intestinal microbial community. By contrast, *B. subtilis* could effectively regulate intestinal microbial community by several competitive exclusion mechanisms (Hong et al., 2005) but could not obtain the same growing performance with the use of AGPs in pullets (Gadde et al., 2017). Hence, the simultaneous short-termed addition of AGP and *B. subtilis* was studied in the present study, which might concurrently maintain the growth performance and the intestinal microbial community. A limitation to the current study is that the biological activity of the *B. subtilis* probiotic alone and together with the AGPs was not confirmed directly. However, *B. subtilis* DSM17299 could effectively survive under acidic condition (pH 2.0), bile salt condition (0.3%), and differential temperature condition (37, 85, and 95°C) and could effectively inhibit the growth of *Salmonella* in broilers (Knap et al., 2011), which proved that B. subtilis DSM17299 has biological activity when it enters the small intestine of the animal and then changes to spore form when it enters the hindgut (Knap et al., 2011). Meanwhile, it was controversial whether the probiotic was resistant to the AGP or was active in the gastrointestinal tract when simultaneously supplemented with the AGPs (Rehman et al., 2012; Chandra et al., 2015). Considering these arguments, the AGPs used in the present study were bacitracin zinc and colistin sulfate. Bacitracin zinc, which is produced by *B. subtilis*, could not completely break the activity of *B. subtilis* in the gut (Eppelmann et al., 2001; Cao and Helmann, 2002; Ohki et al., 2003). Hence, *B. subtilis* is a commercially available product that improves broiler performance to the same extent as an AGP (Knap et al., 2011). Meanwhile, colistin sulfate has bactericidal effects only on gram-negative bacteria (Li et al., 2006); however, *B. subtilis* is a gram-positive bacterium. Overall, *B. subtilis* should be active in the gastrointestinal tract when simultaneously supplemented with bacitracin zinc and colistin sulfate.

The good growth or developmental conditions before laying was beneficial to exert the maximal genetic potential and the excellent laying performance. We focused on the development of some key organs in different stages in the present study, which could represent the growth and developmental conditions of the pullets in different periods. As a result, the body weight of 6-week-old pullets in all groups was up to the standard (about 440 g), and the weight homogeneity was greater than 80%. Whereas, in 7~12 week-old pullets, the tibia length was around 10 cm long in all groups. These results suggested that the short-term simultaneous addition (0~3 weeks) of AGP + *B. subtilis* seemed to be comparable with the long-term use of AGP for the maintenance of growth performance of 0~16 week-old pullets. Meanwhile, numerous studies from the past have shown that *B. subtilis* in various concentrations could promote the growth performance of chickens (Jeong and Kim, 2014; Li et al., 2015; Bai et al., 2017; Gadde et al., 2017; Gao et al., 2017; Jayaraman et al., 2017) when compared with the blank control group. In addition, Gadde et al. (2017) showed that dietary co-supplementation of antibiotic and probiotics (*B. subtilis*) could significantly improve the feed efficiency as evidenced by decreased FCR compared with controls, but, also, there were no differences between AGP and probiotics. Combining our results and the results from previous studies, it could be inferred that the simultaneous supplementation of *B. subtilis* + AGPs in the early stages of broilers (0~3 weeks) could exert similar effects on growth performance when compared with the AGPs group.

Post-hatch changes are more pronounced in the intestine of the chicks when compared with other parts (Prabakar et al., 2016). Spores of *B. subtilis* were induced to germinate either prior to or upon entry into the small intestine of chicks, and they promoted intestinal development (Cartman et al., 2008). Several studies also demonstrated that *B. subtilis* had effects on intestinal morphology, which was similar to the results obtained from the present study (Sen et al., 2012; Al-Fataftah and Abdelqader, 2014). In the present study, dietary co-supplementation of AGP and *B. subtilis* for the first 3 weeks improved intestinal morphology during 7~12 weeks. Upregulated sucrase expression and increased sIgA content in the intestinal mucosa during 13~16 weeks could represent the improved intestinal digestion and absorption functions and intestinal mucosal immunity, respectively (Knap et al., 2011; Wang et al., 2017). These alterations in the gut, especially the improved intestinal morphology, may help the pullets in the BA3 group to obtain similar growth performance as the pullets in the AGPs group.

Previous studies demonstrated that the spores of *B. subtilis* were able to germinate when simultaneously supplemented with bacitracin zinc and colistin sulfate (Eppelmann et al., 2001; Cao and Helmann, 2002; Ohki et al., 2003) and further played the role of a probiotic in the GI tract of chicken (Cartman et al., 2008). However, *B. subtilis* is a transient member of the chick GI microflora, which does not colonize the gastrointestinal tract (Cartman et al., 2008). Hence, the positive effect of simultaneous supplementation of *B. subtilis* and AGPs on the growth performance might be attributed to the establishment of intestinal microbiota at an early period, thus, exerting growth-promoting functions during 7~16 weeks. Our results indicated that the simultaneous supplementation of AGP and *B. subtilis* had better effect on the diversity and homeostasis of intestinal bacteria than AGP alone, according to the PCoA analyses, which was in accordance with the previous results of Rehman et al. (2012). Further analysis of differential genera showed that the relative abundance of *Lactobacillus, Dorea, Ruminococcus*, and *Oscillospira* within the phylum *Firmicutes* were increased in the BA3 group at week 6 (*P* < 0.05). Previous studies showed that *Lactobacillus* was associated with reduced intestinal inflammation (Chen et al., 2012) and enhanced immunological barrier function (Patel et al., 2012). *Dorea* belongs to the *Lachnospiraceae* family, which is related to intestinal inflammation (Videnska et al., 2013) and can prevent intestines disease (Surana and Kasper, 2017). *Ruminococcus* can digest the cellulose in feed and produce short chain fatty acids (SCFAs) through glucose metabolism (Liu et al., 2008; Li et al., 2015), which is beneficial to the gut health. *Oscillospira* was positively correlated with secondary bile acids (BAs) and negatively correlated with primary BAs, which was proven to play important roles in digesting lipids and preventing bacterial infections in the small intestine (Keren et al., 2015). Therefore, simultaneous addition of AGP and *B. subtilis* for 0~3 weeks could increase the proportions of these four caecal beneficial bacteria. Meanwhile, the relative abundances of *Bacteroides, Alistipes, and Odoribacter* within the phylum *Bacteroidetes* in the BA3 group at week 6 were decreased (*P* < 0.05). A recent study showed that the increase in the abundance of these bacteria in *Bacteroidetes* (such as *Alistipes*) were related to increased intestinal disease (Saulnier et al., 2011), which indicated that simultaneous addition of AGP and *B. subtilis* for 0~3 weeks could inhibit the proportions of more than three kinds of caecal bacteria related to inflammation. Overall, our results showed that the simultaneous supplementation of AGP and *B. subtilis* for 0~3 weeks was beneficial for the gut microbiota at week 6. The establishment of caecal microbial community mainly happened in the first 6 weeks (Adi and Magray, 2012), which were the critical periods for the development of microbiota and could be regulated by the simultaneous supplementation of AGP and *B. subtilis* in the early period. Hence, PCoA analyses from the present study also showed that the microbial community in the first 6 weeks was impressionable, and the microbial composition tended to stabilize from 7 to 16 weeks. To sum up, simultaneous supplementation of AGP and *B. subtilis* for 0~3 weeks could mainly play important roles in regulating gut microbiota at week 6 and in further maintaining an altered gut microbiota in the following weeks. Furthermore, results of absolute qPCR at week 12 showed that dietary AGP + *B. subtilis* supplementation for the first 3 weeks elevated the numbers of *B. fragilis* and *C. leptum* in caecum when compared with the AGP group, which were all beneficial bacteria in the gut (Wexler, 2007; Louis and Flint, 2009). These results again revealed that simultaneous supplementation of AGP and *B. subtilis* in the early stage could exert beneficial effect on caecal community in the later period (from 7 to 16 weeks), which was consistent with the results concluded from the 16S rRNA sequencing.

Gut microbiota alteration could influence the intestinal morphology (Louis and Flint, 2009; Jahanian et al., 2017; Vineetha et al., 2017). A negative correlation between the population of caecal *B. fragilis* and ileal crypt depth was identified by further Pearson correlation analysis. Meanwhile, the abundance of caecal *C. leptum* was positively correlated with the duodenal villus height of 6-week-old pullets and the ileac villus height of 12-week-old pullets. These results indicated that *B. fragilis* and *C. leptum* in caecum could improve the development of the small intestine by reducing the crypt depth of ileum and increasing the villus height of ileum and duodenum. In the previous studies, *C. leptum* could produce volatile fatty acid (VFAs) through carbohydrate fermentation (Louis and Flint, 2009), which could maintain gut health and served as the major source of energy for the gut mucosa to promote the growth of gut villus (Louis and Flint, 2009). Meanwhile, polysaccharides produced by *B. fragilis* are important for the activation of the T-cell-dependent immune response, which is beneficial for the gut health (Wexler, 2007). These studies proved that significantly altered *B. fragilis* and *C. leptum* of the present study could actually be beneficial for the development of intestinal morphology. Overall, simultaneous addition of AGP and *B. subtilis* for 0~3 weeks could regulate and stabilize gastrointestinal microbiota in the early stage (0~6 weeks) and improve the intestinal morphology and mucosal immunity by elevating populations of *B. fragilis* and *C. leptum* in caecum in the later period (7~16 weeks), to maintain the overall growth and development of the pullets.

## Conclusion

Simultaneous supplementation of AGP and *B. subtilis* for 0~3 weeks, firstly, increased the relative abundance of beneficial microbiota in caecum in 0~6-week-old pullets and then further maintained the overall growth and development of pullets by promoting intestinal morphology and by upregulating intestinal sucrase expression during 7~16 weeks. The improved intestinal morphology was positively correlated with the elevated populations of *B. fragilis* and *C. leptum*. Overall, our results indicated that the simultaneous short-termed supplementation of AGP and *B. subtilis* at early developmental stages of pullets could be beneficial for the construction of salutary intestinal microbiota and the early growth and development of pullets.

## Acceptable statements

The raw data supporting the conclusions of this manuscript will be made available by the authors, without undue reservation, to any qualified researcher.

## Author contributions

XYL, SW, XL, and TY designed the research; XYL, SW, XL, TY, YD, XY, QS, YLD and XJY performed the research and analyzed the data; SW, XYL and XL wrote the manuscript; SW, XYL, and XL have taken part in the revision of the manuscript. All authors read and approved the final version of the manuscript.

### Conflict of interest statement

The authors declare that the research was conducted in the absence of any commercial or financial relationships that could be construed as a potential conflict of interest.
