# Supplementary material for: Simultaneous Supplementation of Bacillus subtilis and Antibiotic Growth Promoters by Stages Improved Intestinal Function of Pullets by Altering Gut Microbiota
Source: Front Microbiol. 2018 Oct 12;9:2328. doi: 10.3389/fmicb.2018.02328 (PMC6194165; doi:10.3389/fmicb.2018.02328)
Supplement: Supplementary file 1 [file Table_1.DOCX]

**Simultaneous supplementation of Bacillus Subtilis and Antibiotic Growth Promoters by Stages Improved Intestinal Function of Pullets by Altering Gut Microbiota**

Xueyuan Li^#, †^ , Shengru Wu ^#,^ *^, †^, Xinyi Li^#, †^ ,Tao Yan^#^, Yongle Duan^#^, Xin Yang^#^, Yulan Duan^#^, Qingzhu Sun^#^, Xiaojun Yang^#, 1^

**Correspondence:** Xiaojun Yang: [yangxj@nwsuaf.edu.cn](mailto:yangxj@nwsuaf.edu.cn)

**Supplementary files**

**Supplementary** **Figure 1.** The standard curve and equation of *C. leptum*, *B. fragilis*, *E. coli*, and *S. enteritidis* plasmids. Note: The abscissa represented lg vaules of copy numbers in the plasmids by dilutions (10^-1^~10^-6^) and the ordinate represented the cycle threshold vaules, R^2^ > 0.99.

**Supplementary** **Figure 2** Abundance of the predicted gene related to KEGG pathways at level 1 and 2 at 3 week. Note: Yellow box: AGP samples, purple box: BA3 samples. The terms given on the left are KEGG pathways annotation at level 1 and level 2 (from left to right).

**Supplementary** **Figure 3** Abundance of the predicted gene related to KEGG pathways at level 1 and 2 at 6 week. Note: Purple box: AGP samples, yellow box: BA3 samples, blue box: BA6 samples. The terms given on the left are KEGG pathways annotation at level 1 and level 2 (from left to right).

**Supplementary** **Figure 4** Abundance of the predicted gene related to KEGG pathways at level 1 and 2 at 12 week. Note: Blue box: AGP samples, red box: BA3 samples, yellow box: BA6 samples,dark blue box: BA12 samples. The terms given on the left are KEGG pathways annotation at level 1 and level 2 (from left to right).

**Supplementary** **Figure 5** Abundance of the predicted gene related to KEGG pathways at level 1 and 2 at 16 week. Note: Blue box: AGP samples, purple box: BA3 samples, yellow box: BA6 samples, dark blue box: BA12 samples, red box: BA16 samples. The terms given on the left are KEGG pathways annotation at level 1 and level 2 (from left to right).

**
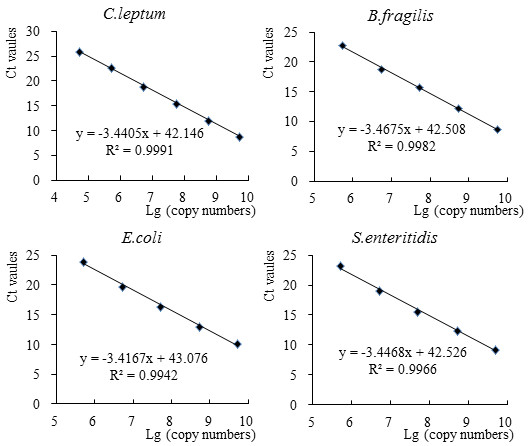
**

**Supplementary Figure 1.** The standard curve and equation of *C.leptum*, *B.fragilis*, *E.coli*, and *S.enteritidis* plasmids. The abscissa represented lg vaules of copy numbers in the plasmids by dilutions (10^-1^~10^-6^) and the ordinate represented the cycle threshold vaules, R^2^>0.99.


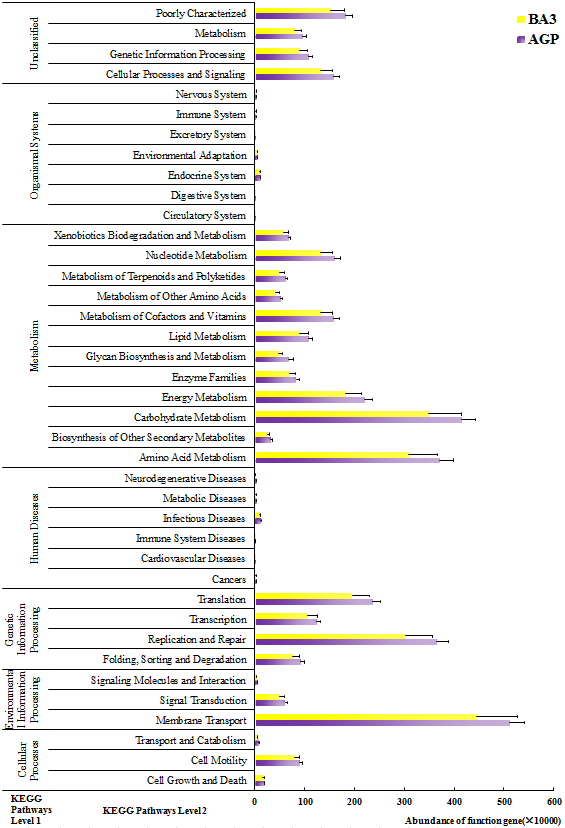
**Supplementary Figure 2.** Abundance of the predicted gene related to KEGG pathways at level 1 and 2 at 3 week. Yellow box: AGP samples, purple box: BA3 samples. The terms given on the left are KEGG pathways annotation at level 1 and level 2 (from left to right).

**
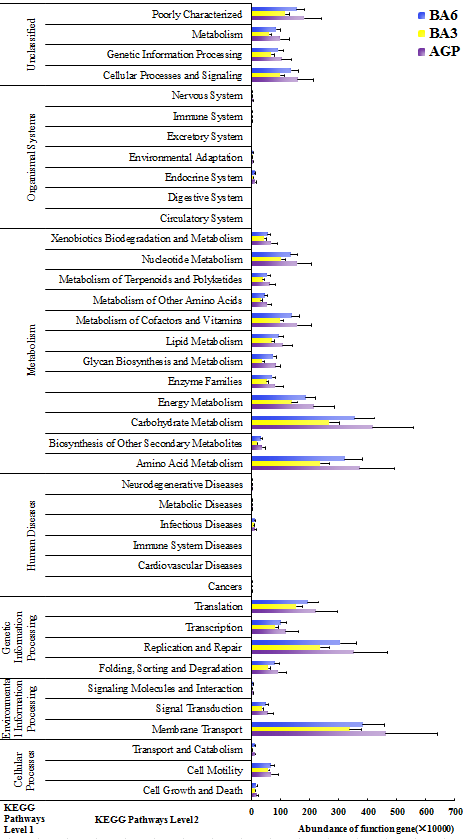
Supplementary** **Figure 3.** Abundance of the predicted gene related to KEGG pathways at level 1 and 2 at 6 week. Purple box: AGP samples, yellow box: BA3 samples, blue box: BA6 samples. The terms given on the left are KEGG pathways annotation at level 1 and level 2 (from left to right).


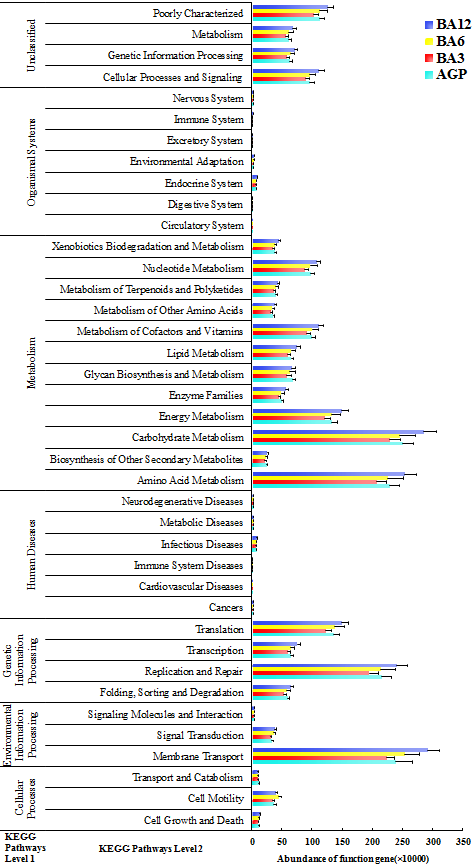
**Supplementary** **Figure 4.** Abundance of the predicted gene related to KEGG pathways at level 1 and 2 at 12 week. Blue box: AGP samples, red box: BA3 samples, yellow box: BA6 samples,dark blue box: BA12 samples. The terms given on the left are KEGG pathways annotation at level 1 and level 2 (from left to right).

**
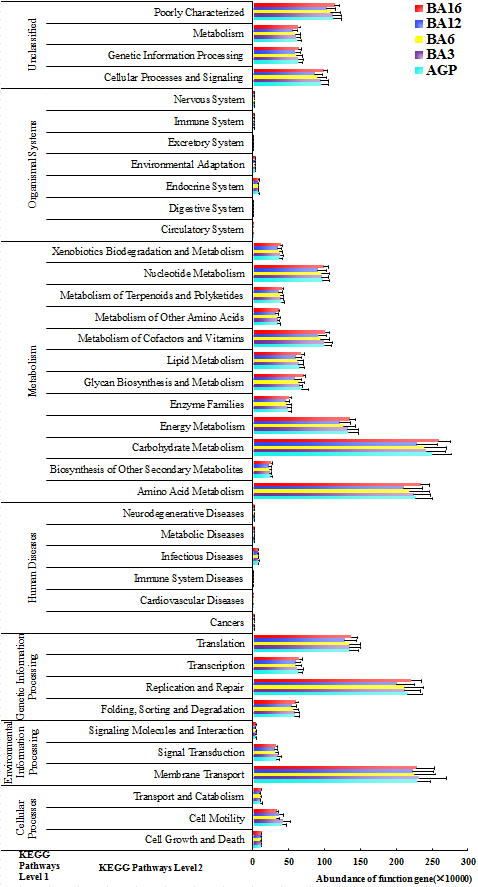
Supplementary** **Figure 5.** Abundance of the predicted gene related to KEGG pathways at level 1 and 2 at 16 week. Blue box: AGP samples, purple box: BA3 samples, yellow box: BA6 samples, dark blue box: BA12 samples, red box: BA16 samples. The terms given on the left are KEGG pathways annotation at level 1 and level 2 (from left to right).
